# Supplementary material for: Unusual facet and co-catalyst effects in TiO2-based photocatalytic coupling of methane
Source: Nat Commun. 2024 May 24;15:4453. doi: 10.1038/s41467-024-48866-1 (PMC11126583; doi:10.1038/s41467-024-48866-1)
Supplement: Supplementary file 1 — Supplementray Information [file 41467_2024_48866_MOESM1_ESM.pdf]

Supplementary Information

Unusual facet and co-catalyst effects in TiO_2-based photocatalytic coupling of methane

Huizhen Zhang[1,3], Pengfei Sun[1,3], Xiaozhen Fei[1], Xuejiao Wu[1], Zongyi Huang[1], Wanfu Zhong[1], Qiaobin Gong[1], Yanping Zheng[1], Qinghong Zhang[1], Shunji Xie[1,2]*, Gang Fu[1,2]* and Ye Wang[1,2]*

[1]State Key Laboratory of Physical Chemistry of Solid Surfaces, Collaborative Innovation Center of Chemistry for Energy Materials, National Engineering Laboratory for Green Chemical Productions of Alcohols, Ethers and Esters, College of Chemistry and Chemical Engineering, Xiamen University, Xiamen 361005, China.

[2]Innovation Laboratory for Sciences and Technologies of Energy Materials of Fujian Province (IKKEM), Xiamen 361005, China.

[3]These authors contributed equally: Huizhen Zhang, Pengfei Sun.

*e-mail: shunji_xie @xmu.edu.cn; gfu@xmu.edu.cn; wangye@xmu.edu.cn

Supplementary Figures

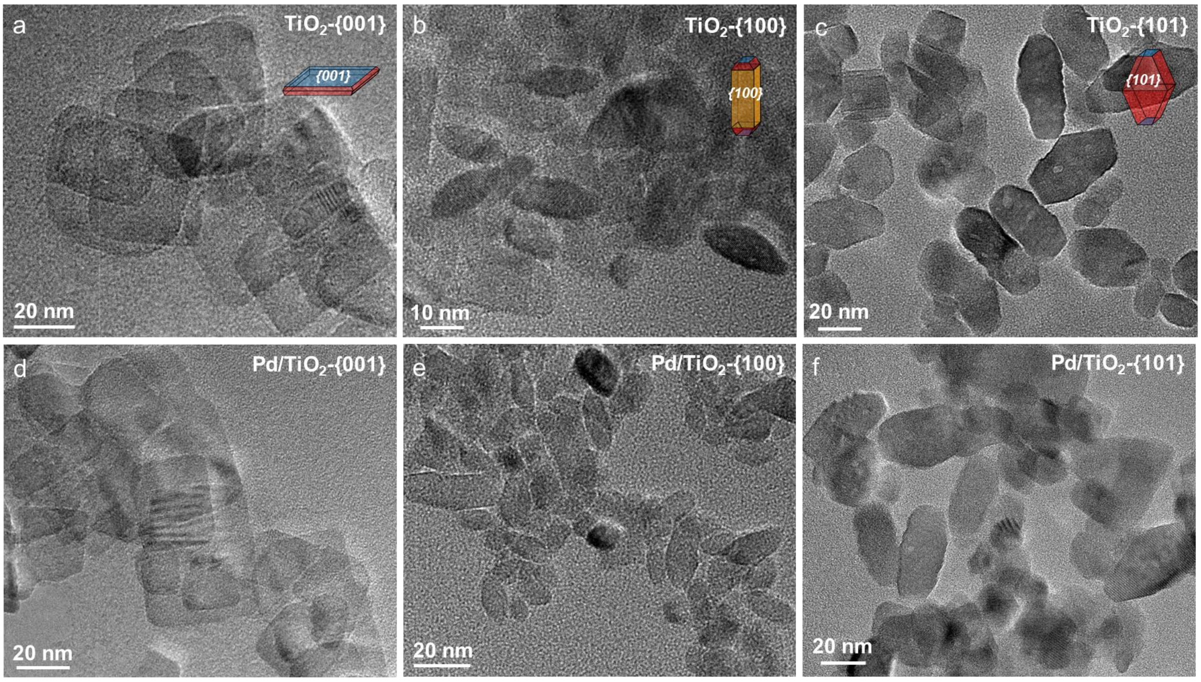

Supplementary Fig. 1 | Morphologies of TiO_2 and Pd/TiO_2 catalysts. a, TEM image of TiO_2-{001}. **b,** TEM image of TiO_2-{100}. **c,** TEM image of TiO_2-{101}. **d,** TEM image of Pd/TiO_2-{001}. **e,** TEM image of Pd/TiO_2-{100}. **f,** TEM image of Pd/TiO_2-{101}.

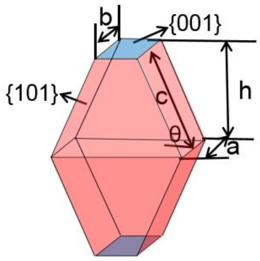

a=25 nm; b=9 nm; h=24 nm; θ=68.3º

$c=h/\sin\theta=24/0.929=25.8$

$S_{\{001\}}=2*b^2=162$

$S_{\{101\}}=8*(a+b)*c/2=3508$

$P_{\{101\}}=S_{\{101\}}/(S_{\{001\}}+S_{\{101\}})=95\%$

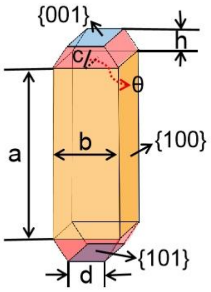

a=30 nm; b=10 nm; d=6 nm; h=3 nm; θ=68.3º

$c=h/\sin\theta=3/0.929=3.2$

$S_{\{001\}}=2*d^2=72$

$S_{\{101\}}=8*(b+d)*c/2=204$

$S_{\{100\}}=4*a*b=1200$

$P_{\{100\}}=S_{\{100\}}/(S_{\{100\}}+S_{\{001\}}+S_{\{101\}})=81\%$

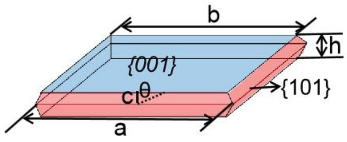

a=38 nm; b=33 nm; h=3.4 nm; θ=68.3º

$c=h/(2*\sin\theta)=3.4/(2*0.929)=1.8$

$S_{\{001\}}=2*b^2=2178$

$S_{\{101\}}=8*(a+b)*c/2=511$

$P_{\{001\}}=S_{\{001\}}/(S_{\{001\}}+S_{\{101\}})=81\%$

Supplementary Fig. 2 | Evaluation of the percentage of major exposed facet for the three TiO_2 nanocrystals. The independent length parameters were obtained from TEM and HRTEM results (Fig. 1 and Supplementary Fig. 1). Note that θ is the theoretical value (68.3º) of the angle between the {101} and {001} facets of anatase TiO_2[1].

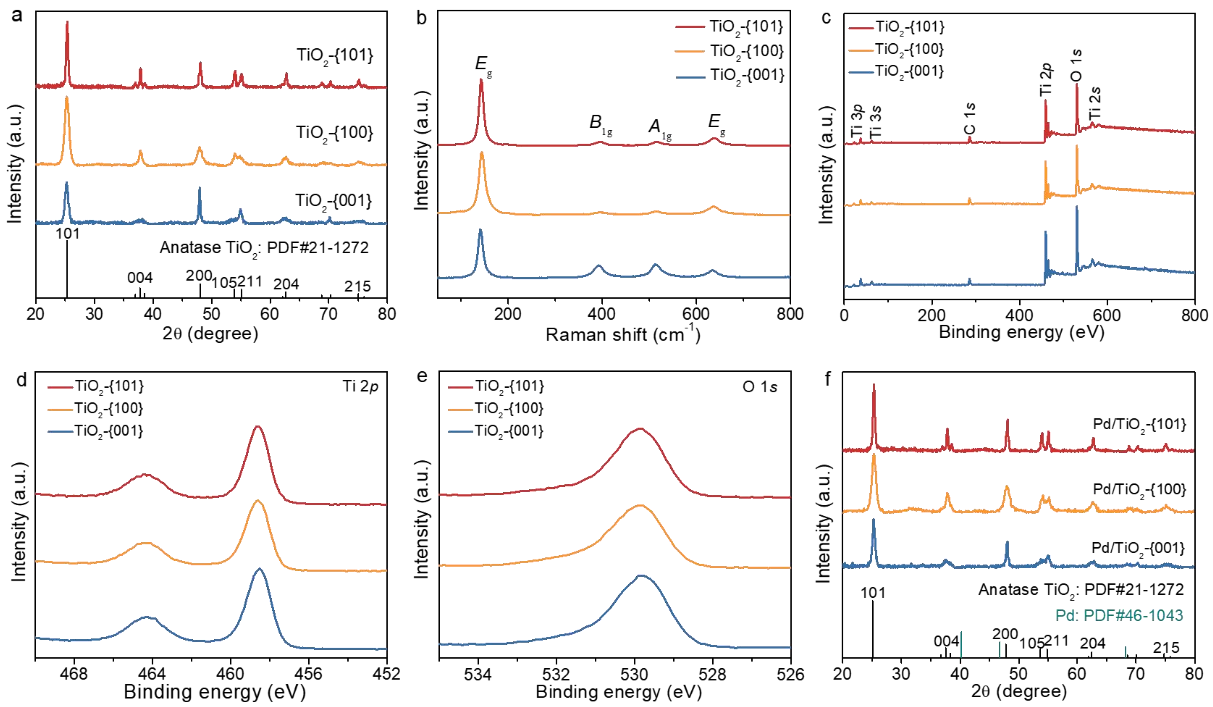

Supplementary Fig. 3 | Characterizations of crystalline and surface structures. a, XRD patterns. **b,** Raman spectra. **c,** XPS survey spectra. **d,** Ti 2*p* XPS spectra. **e,** O 1*s* XPS spectra. **f,** XRD patterns. The signal of C 1*s* in XPS survey spectra originates from the contaminant carbon, which is typically used as the standard for calibration of binding energy values.

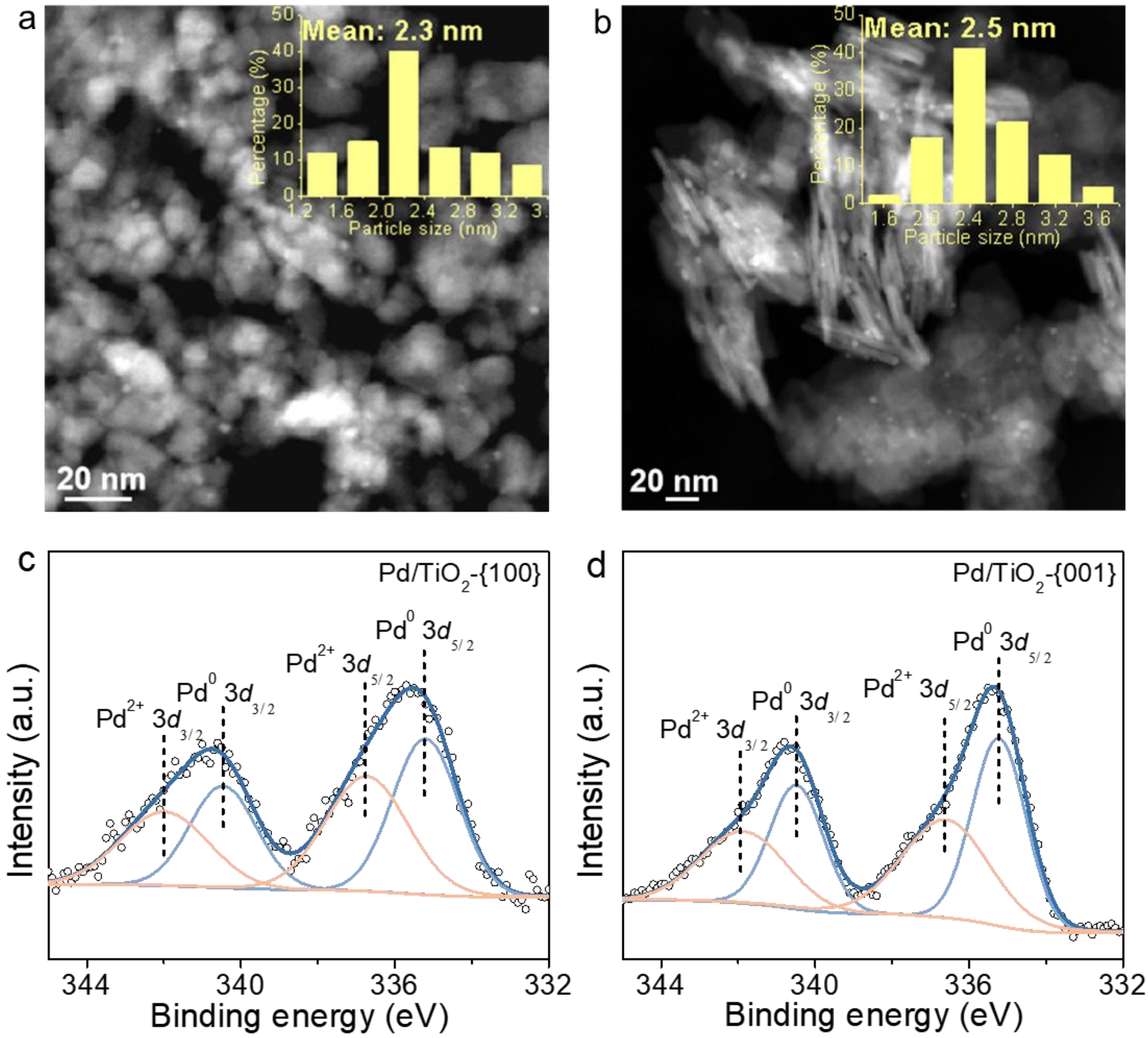

Supplementary Fig. 4 | STEM images and XPS spectra. a, STEM image and Pd size distribution (inset) for Pd/TiO_2-{100}, **b,** STEM image and Pd size distribution (inset) for Pd/TiO_2-{001}. **c,** Pd 3*d* XPS spectrum for Pd/TiO_2-{100}. **d,** Pd 3*d* XPS spectrum for Pd/TiO_2-{001}.

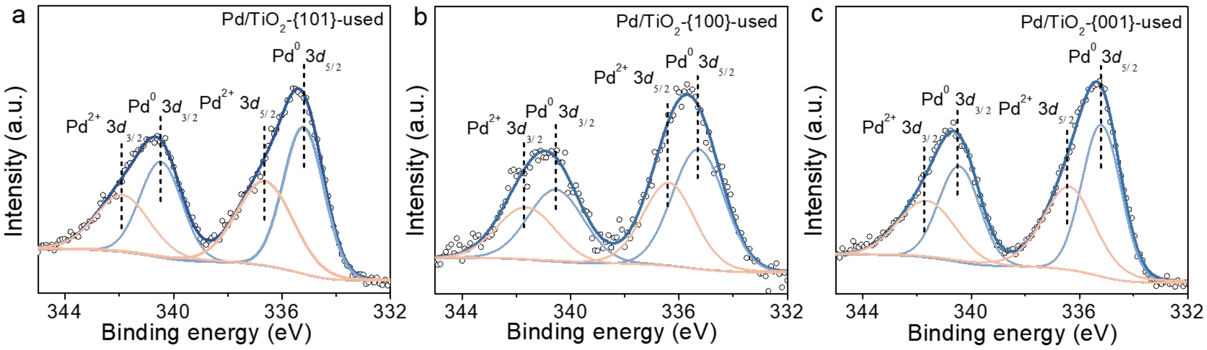

Supplementary Fig. 5 | Pd XPS spectra for the Pd/TiO_2 catalysts after the photocatalytic NOCM reaction. a, Pd/TiO_2-{101}-used. **b,** Pd/TiO_2-{100}-used. **c,** Pd/TiO_2-{001}-used. Reaction conditions: catalyst, 20 mg; water, 50 mL; CH_4, 45 mL (2009 μmol); light source, 300 W Xe lamp (λ = 320-780 nm); irradiation time, 4 h.

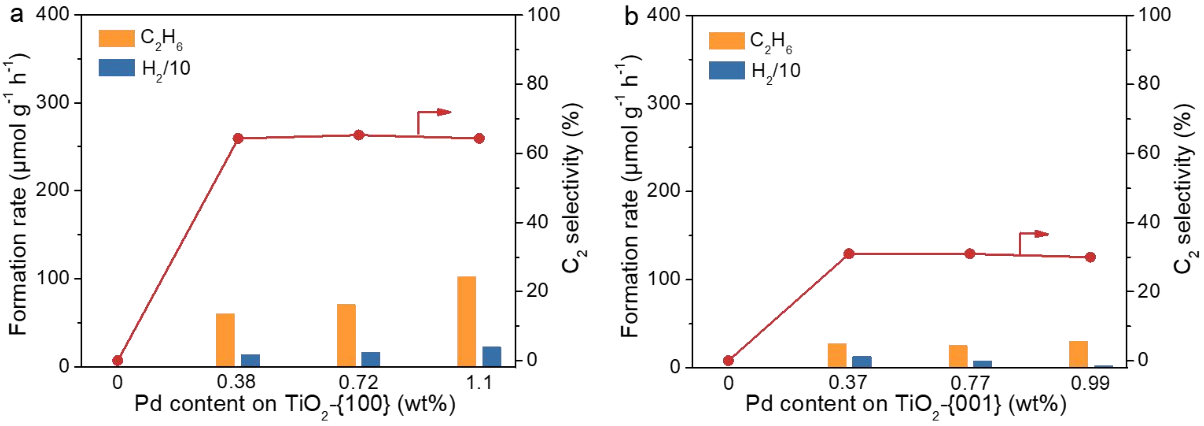

Supplementary Fig. 6 | Effect of Pd content on photocatalytic NOCM performances. a, Pd/TiO_2-{100}. **b,** Pd/TiO_2-{001}.

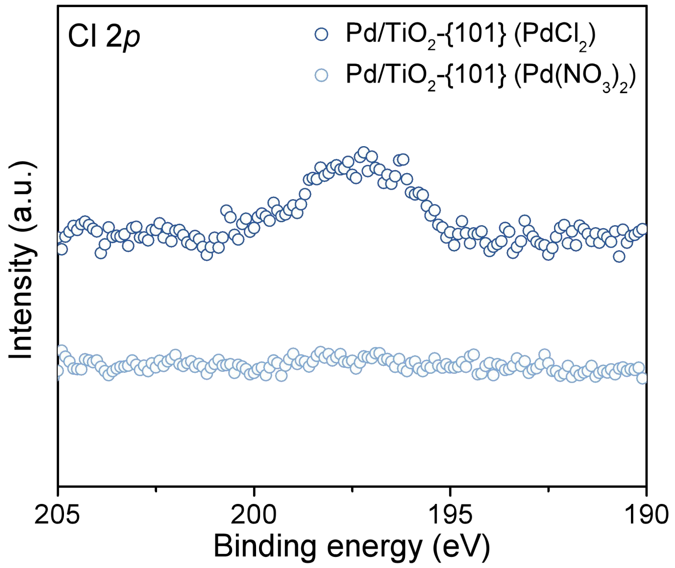

Supplementary Fig. 7 | Cl 2*p* XPS spectra for Pd-loaded TiO_2-{101} catalysts. $PdCl_2$ or $Pd(NO_3)_2$ was used as the Pd precursor.

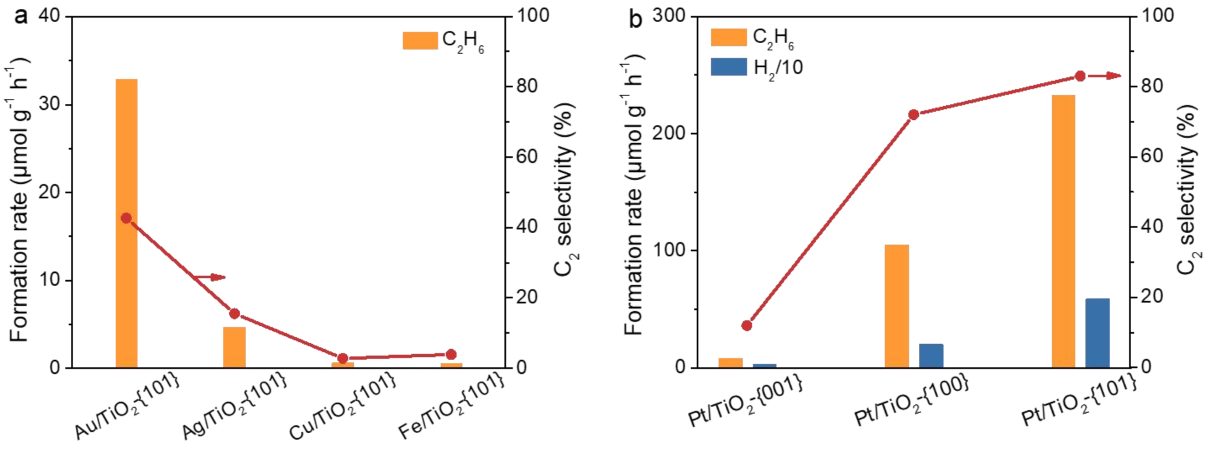

Supplementary Fig. 8 | Photocatalytic NOCM performances for other metal-loaded TiO_2-{101} catalysts. a, Performances of Au-, Ag-, Cu-, and Fe-loaded TiO_2-{101}. **b,** Performances of Pt-loaded TiO_2 catalysts with different exposed anatase facets.

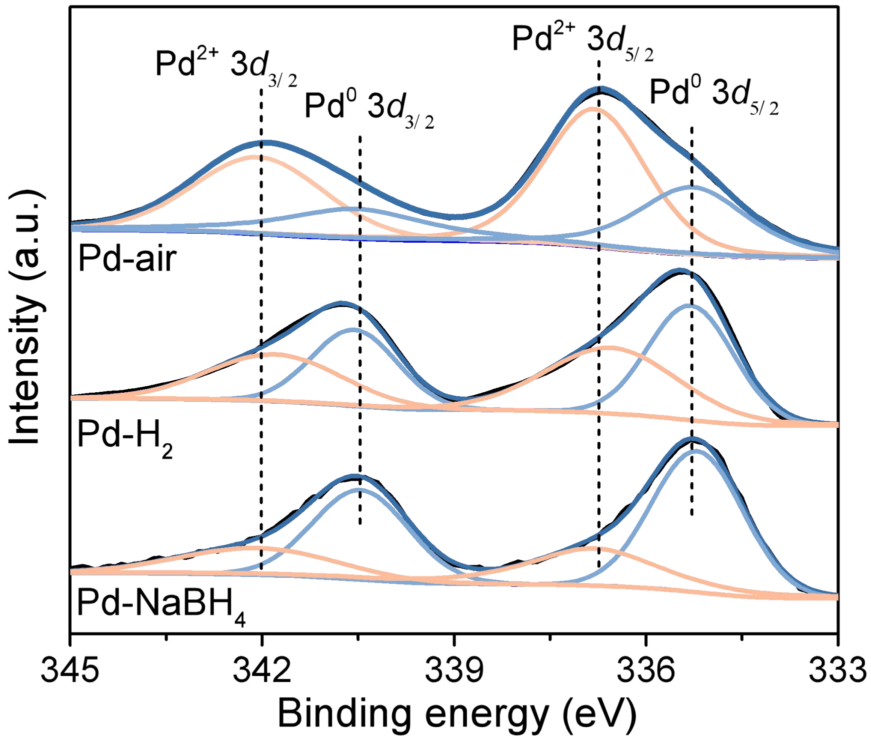

Supplementary Fig. 9 | Pd 3*d* XPS spectra for Pd/TiO_2-{101} catalysts. The catalysts were prepared by different post-treatments of the $PdCl_2$/TiO_2 precursor.

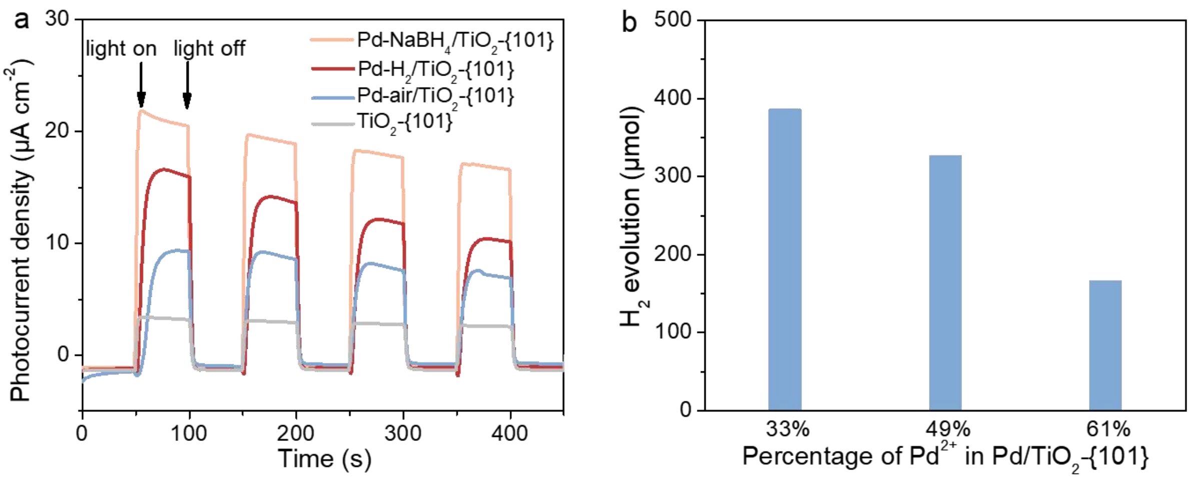

Supplementary Fig. 10 | Transient photocurrent responses and H_2 evolution for Pd/TiO_2-{101} catalysts. The catalysts were prepared by different post-treatments of the $PdCl_2/TiO_2$ precursor. **a**, Transient photocurrent responses. **b**, Photocatalytic H_2 evolution. For H_2 evolution reactions, the catalyst (0.0050 g) was dispersed in a mixed solution of methanol (2 mL) and water (3 mL). The suspension was irradiated for 2 h under 300 W Xe lamp (λ = 320-780 nm) under N_2.

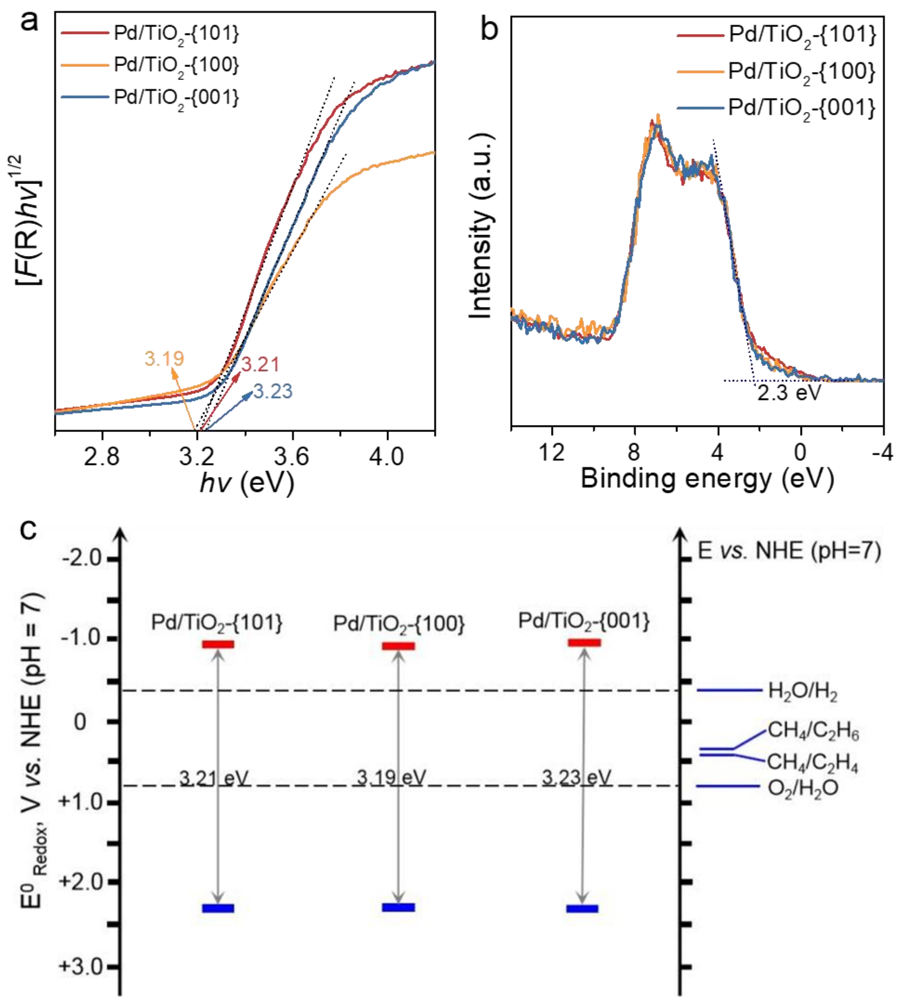

Supplementary Fig. 11 | Energy band structures of Pd/TiO_2 catalysts with different exposed anatase facets. a, Plots of modified Kubelka-Munk function versus the energy of exciting light. **b**, XPS valence spectra. **c**, Energy levels.

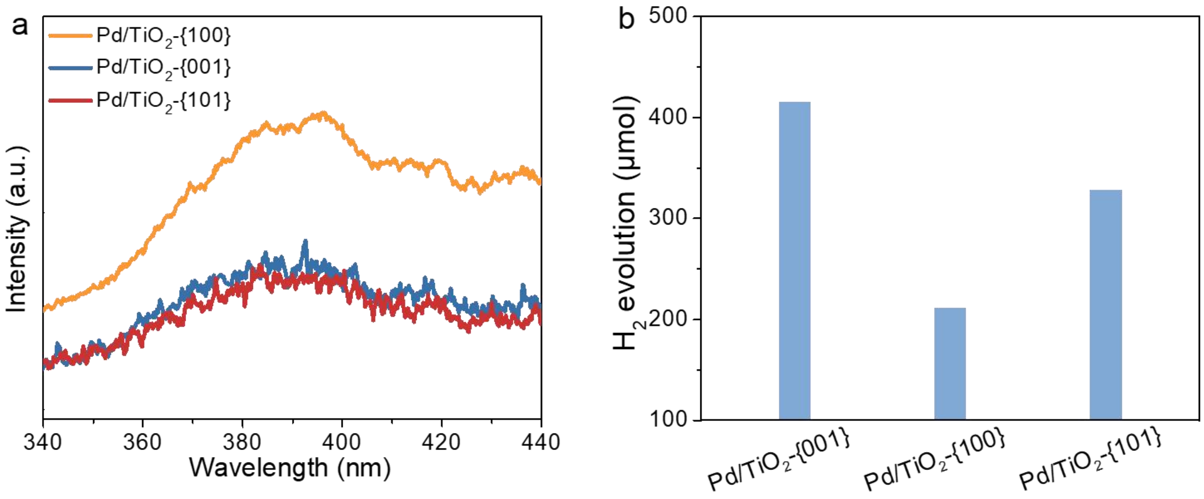

Supplementary Fig. 12 | Photoluminescence spectra and H_2 evolution for Pd/TiO_2 catalysts with different exposed anatase facets. **a**, Photoluminescence spectra at an excitation wavelength of 280 nm. **b**, Photocatalytic H_2 evolution. For H_2 evolution reactions, the catalyst (0.0050 g) was dispersed in a mixed solution of methanol (2 mL) and water (3 mL). The suspension was irradiated for 2 h under 300 W Xe lamp (λ = 320-780 nm) under N_2.

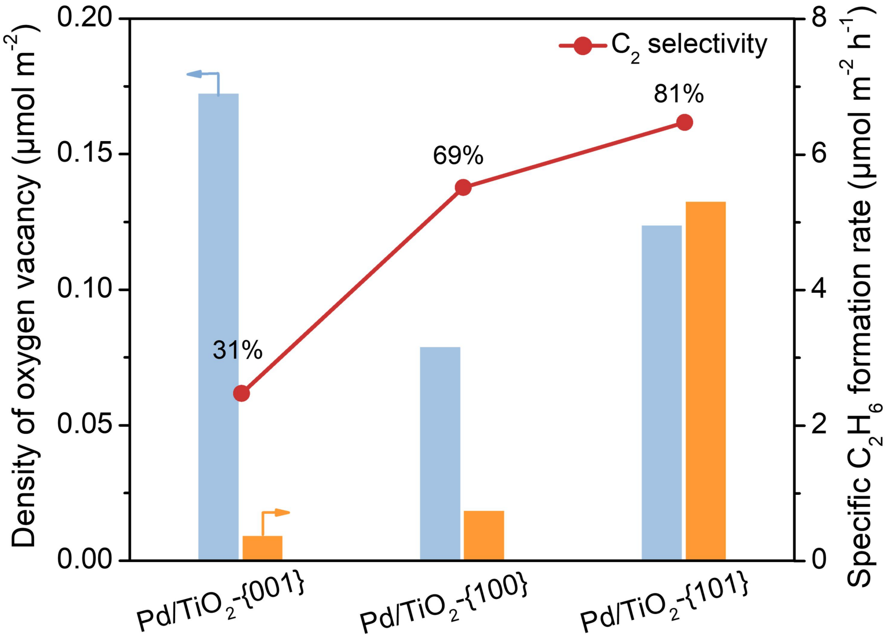

Supplementary Fig. 13 | Density of oxygen vacancies and photocatalytic performance versus exposed facet.

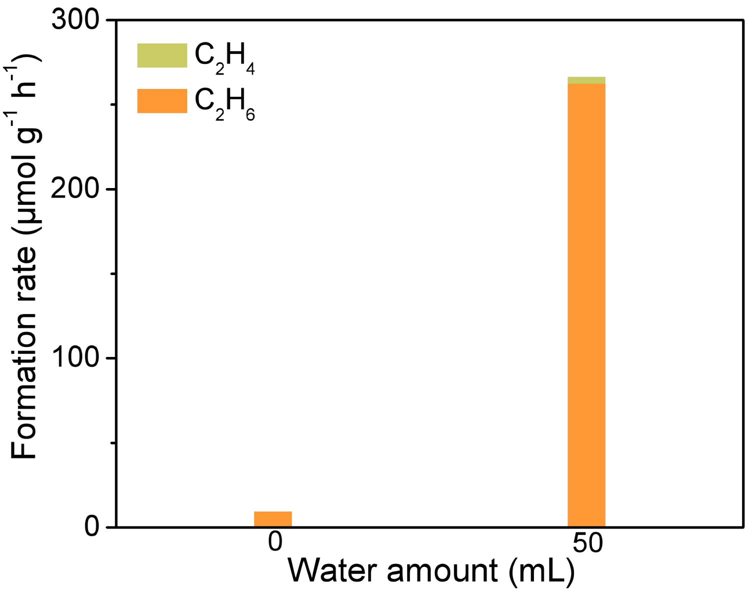

Supplementary Fig. 14 | Effect of water on photocatalytic NOCM performances.

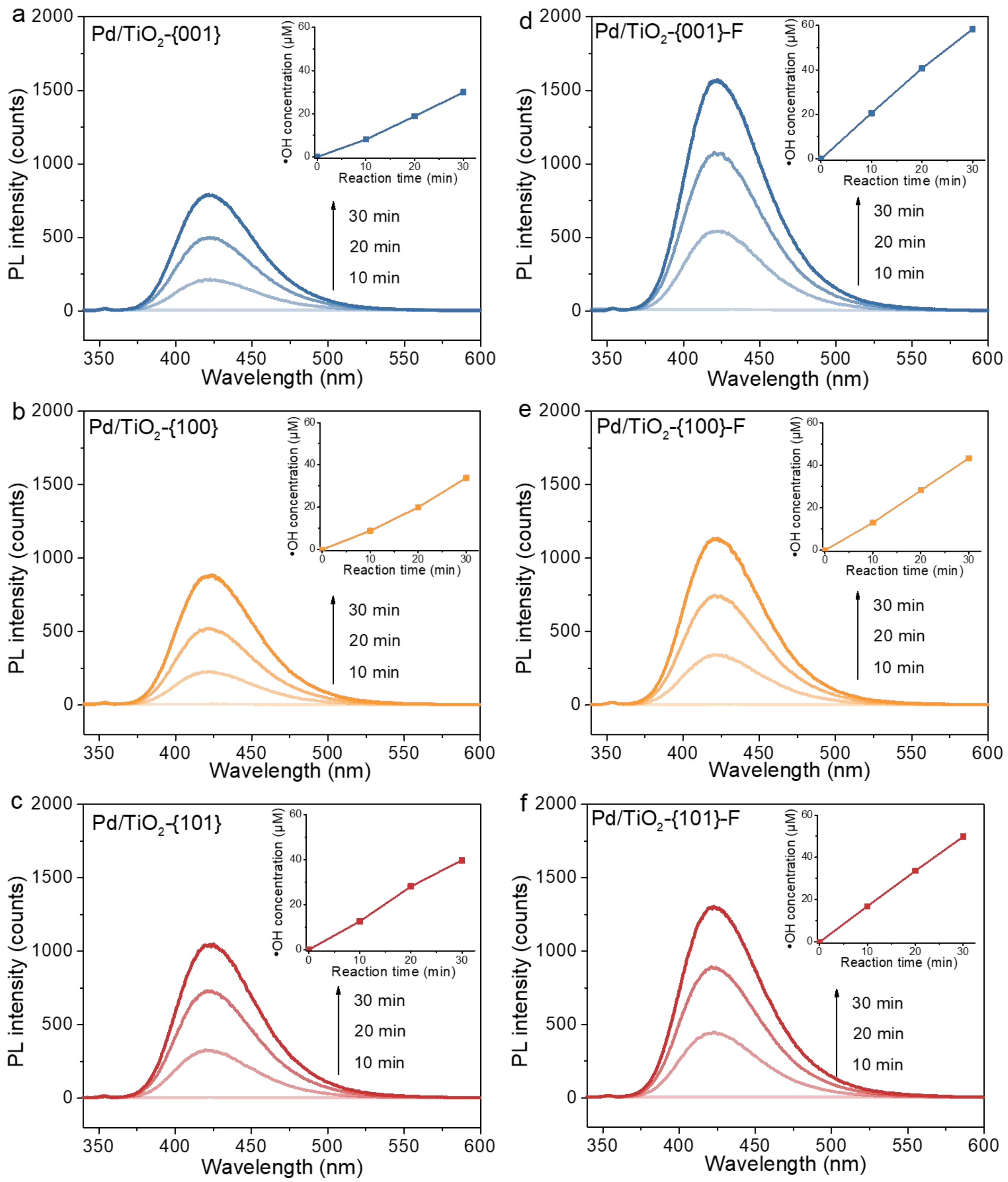

Supplementary Fig. 15 | Quantification of •OH radicals by photoluminescence spectroscopy. a-f, Photoluminescence (PL) intensity corresponding to •OH radicals for Pd/TiO_2-{001}, Pd/TiO_2-{100}, Pd/TiO_2-{101}, Pd/TiO_2-{001}-F, Pd/TiO_2-{100}-F, and Pd/TiO_2-{101}-F. The insets of **Supplementary Fig. 15 a-f** display the correlation between the concentration of •OH and the irradiation time.

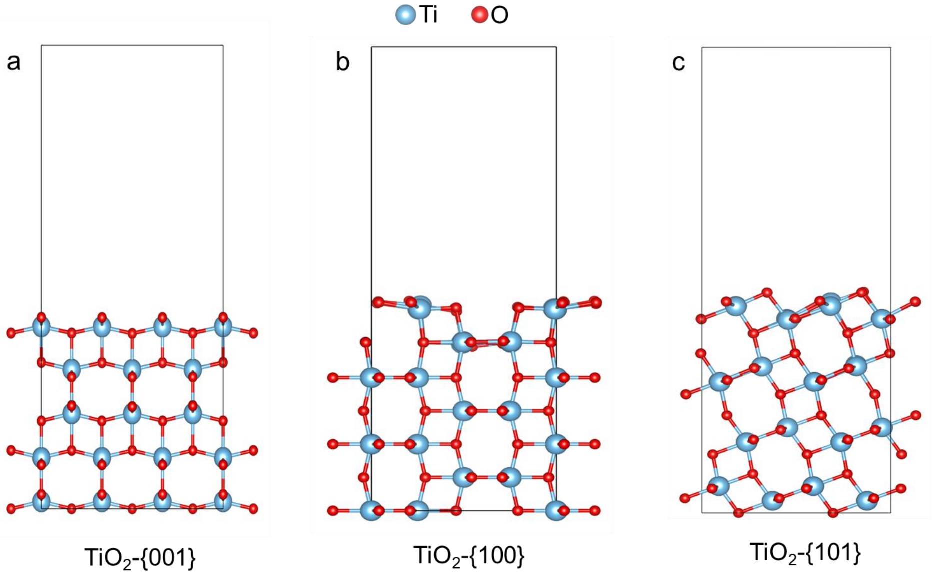

Supplementary Fig. 16 | Slab models for anatase TiO_2 surfaces. a, TiO_2-{001}. **b**, TiO_2-{100}. **c**, TiO_2-{101}.

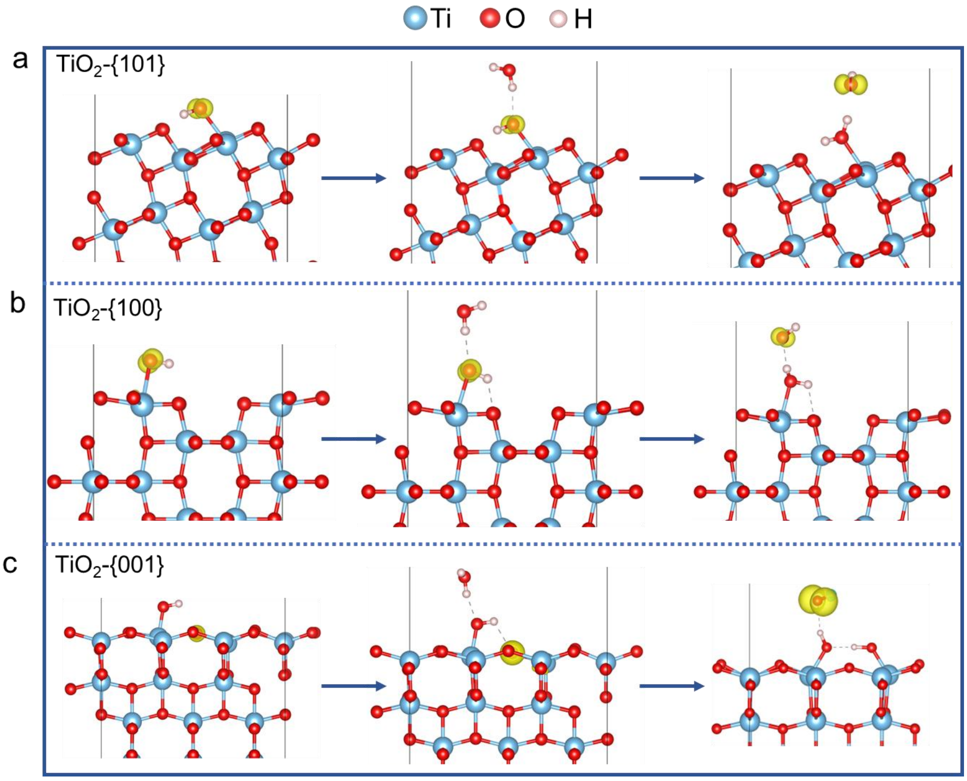

Supplementary Fig. 17 | Optimized structures for •$OH_{liquid\text{-}phase}$ generation by H-transfer.

a, TiO_2-{101} surface. **b,** TiO_2-{100} surface. **c,** TiO_2-{001} surface.

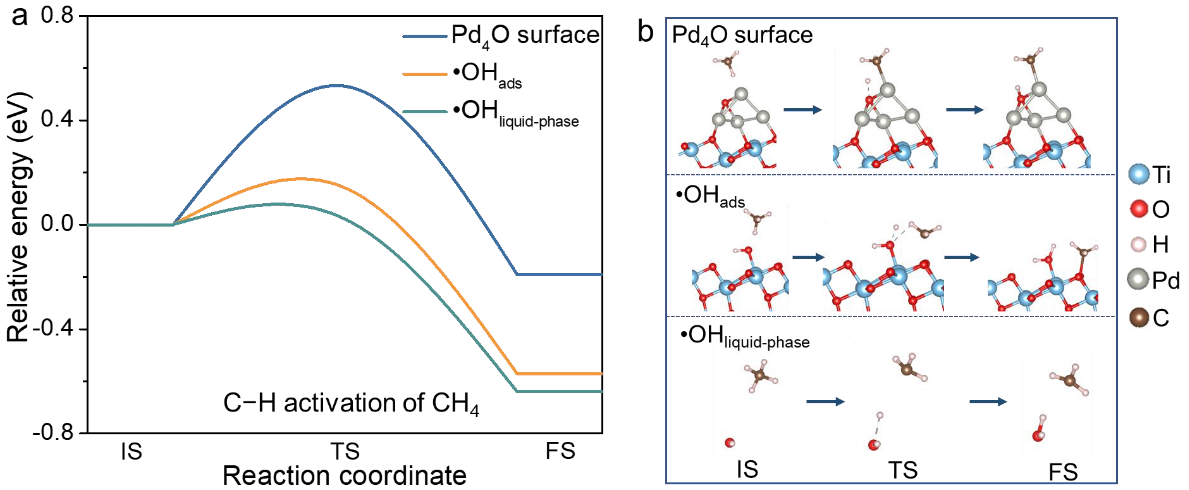

Supplementary Fig. 18 | DFT calculations for the cleavage of the C−H bond in CH_4. a, The relative energies for the cleavage of the C−H bond in CH_4 to $•CH_3$ on Pd_4O cluster over TiO_2-{101} surface, by •OH adsorbed on the TiO_2-{101} surface, and by •OH radicals in the liquid phase. **b,** Structures of the initial state (IS), transition state (TS), and final state (FS) in the CH_4 activation process.

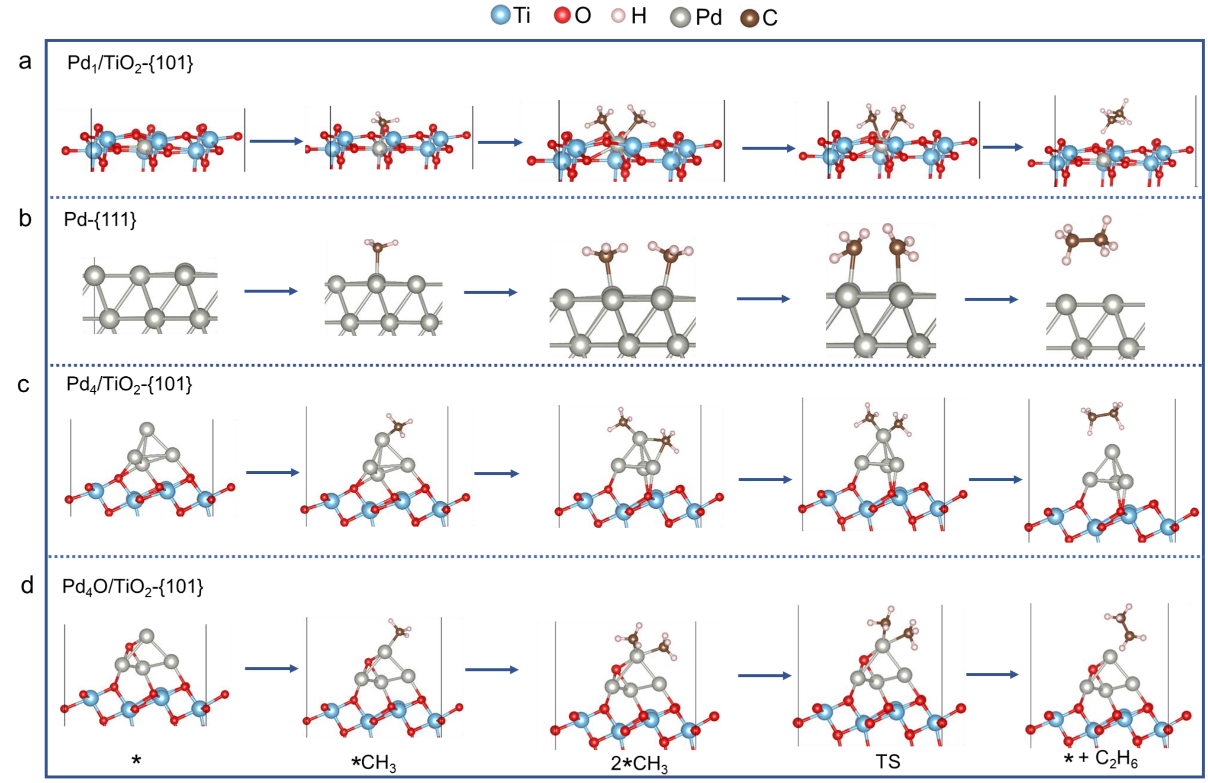

Supplementary Fig. 19 | Optimized structures for •CH_3 enrichment and self-coupling on Pd sites. a, Pd_1/TiO_2-{101}. **b,** Pd-{111}. **c,** Pd_4/TiO_2-{101}. **d,** Pd_4O/TiO_2-{101}.

Supplementary Table 1 | Representative results for the photocatalytic NOCM reaction reported to date.

Catalyst	CH_4 conversion rate (μmol g^{-1} h^{-1})	C_{2+} formation rate (μmol g^{-1} h^{-1})	C_{2+} formation rate (μmol h^{-1})	C_{2+} selectivity (%)	Ref.
Zn^{2+}-ZSM-5	9.8	C_2H_6, 3.0	C_2H_6, 3.0	99	S2
Ga^{3+}-ETS-10	30	C_2H_4, 0.6<br>C_2H_6, 11	C_2H_4, 0.12<br>C_2H_6, 2.2	~100	S3
Au/ZnO	24	C_2H_6, 12	C_2H_6, 0.012	~100	S4
Pt/TiO_2	138	C_2H_6, 51<br>C_2H_4, 2.2	C_2H_6, 3.9<br>C_2H_4, 0.17	62	S5
Pt-TiO_2-SiO_2	3.5	C_2H_6, 1.6	C_2H_6, 0.32	90	S6
Ag-HPW-TiO_2	55	C_2H_6, 23<br>C_3H_8, 1.2	C_2H_6, 2.3<br>C_3H_8, 0.12	90	S7
ZnO-$AuPd_{2.7}$	79	C_2H_6, 25<br>C_2H_4, 13	C_2H_6, 0.05<br>C_2H_4, 0.026	96	S8
Nb-TiO_2-SiO_2	3.6	C_2H_6, 1.7<br>C_3H_8, 0.07	C_2H_6, 0.17<br>C_3H_8, 0.007	96	S9
0.2Pt@BT-O	41	C_2H_4, 0.05<br>C_2H_6, 1.7<br>C_3H_6, 0.55<br>C_3H_8, 8.6<br>C_4H_8, 0.05<br>C_4H_{10}, 1.7<br>> C_4, 0.15	C_2H_4, 0.002<br>C_2H_6, 0.085<br>C_3H_6, 0.028<br>C_3H_8, 0.43<br>C_4H_8, 0.002<br>C_4H_{10}, 0.085<br>> C_4, 0.0075	99	S10
Pd_1/TiO_2	968	C_2H_6, 910	C_2H_6, 2.7	94	S11
Pd/TiO_2-{101}	326	C_2H_6, 262<br>C_2H_4, 4.0	C_2H_6, 5.2<br>C_2H_4, 0.08	81	This work

Supplementary Table 2 | Fractions of Pd^0 and Pd^{2+} on Pd/TiO_2 catalysts measured by XPS as well as the Pd contents.

Catalyst	Pd content[a] (wt%)	Pd^0		Pd^{2+}	
		Binding energy (eV)	Fraction (%)	Binding energy (eV)	Fraction (%)
Pd/TiO_2-{101}	0.78	335.3	51.1	336.6	48.9
Pd/TiO_2-{100}	0.72	335.2	51.3	336.7	48.6
Pd/TiO_2-{001}	0.77	335.2	52.3	336.6	47.7

[a] Pd content was measured by inductively coupled plasma optical emission spectrometer (ICP-OES).

Supplementary Table 3 | Fractions of Pd^0 and Pd^{2+} on Pd/TiO_2 catalysts after photocatalytic NOCM reactions measured by XPS.

Catalyst	Pd^0		Pd^{2+}	
	Binding energy (eV)	Fraction (%)	Binding energy (eV)	Fraction (%)
Pd/TiO_2-{101}-used	335.2	54.6	336.7	45.4
Pd/TiO_2-{100}-used	335.3	57.6	336.5	42.4
Pd/TiO_2-{001}-used	335.2	54.8	336.5	45.2

Reaction conditions: catalyst, 20 mg; water, 50 mL; CH_4, 45 mL (2009 μmol); light source, 300 W Xe lamp (λ = 320-780 nm); irradiation time, 4 h.

Supplementary Table 4 | Photocatalytic NOCM performances.

Catalyst	Product amount (μmol)				CH_4 conversion[a] (%)	C_2 selectivity[b] (%)	C_2 yield[c] (%)
	C_2H_6	C_2H_4	CO_2	H_2			
Pd/TiO_2-{101}	10	0.16	4.8	42	1.3	81	1.1
Pd/TiO_2-{100}	2.8	0	2.5	13	0.40	69	0.27
Pd/TiO_2-{001}	1.0	0	4.2	6.4	0.30	31	0.094
Pd/P25	8.1	0.26	13	61	1.4	56	0.77
Pd/TiO_2-{101}[d]	11	0	5.3	40	1.4	81	1.1
TiO_2-{101} + Pd/Al_2O_3	2.3	0	4.0	12	0.43	53	0.23

Reaction conditions: catalyst, 20 mg; water, 50 mL; CH_4, 45 mL (2009 μmol); light source, 300 W Xe lamp (λ = 320-780 nm); irradiation time, 4 h.

[a] CH_4 conversion = $[2 \times n(C_2H_6) + 2 \times n(C_2H_4) + n(CO_2)] / n(CH_4)$

[b] C_2 selectivity = $[2 \times n(C_2H_6) + 2 \times n(C_2H_4)] / [2 \times n(C_2H_6) + 2 \times n(C_2H_4) + n(CO_2)]$

[c] C_2 yield = CH_4 conversion × C_2 selectivity

[d] $Pd(NO_3)_2$ as the Pd precursor

Supplementary Table 5 | Control experimental results.

Gas atmosphere	Catalyst	Light	Product amount (μmol)			
			C_2H_6	C_2H_4	CO_2	H_2
N_2	Pd/TiO_2-{101}	Yes	0	0	0	2.8
N_2	Pd/TiO_2-{100}	Yes	0	0	0	3.8
N_2	Pd/TiO_2-{001}	Yes	0	0	0	4.0
CH_4	Pd/TiO_2-{101}	No	0	0	0	0
CH_4	Pd/TiO_2-{100}	No	0	0	0	0
CH_4	Pd/TiO_2-{001}	No	0	0	0	0
CH_4	No	Yes	0	0	0	0

Original reaction conditions: catalyst, 20 mg; water, 50 mL; CH_4, 45 mL (2009 μmol); light source, 300 W Xe lamp (λ = 320-780 nm); irradiation time, 4 h.

Supplementary Table 6 | Specific surface areas and specific C_2H_6 formation rates.

Catalyst	Surface area (m^2 g^{-1})	Formation rate of C_2H_6 (μmol g^{-1} h^{-1})	Specific C_2H_6 formation rate (μmol m^{-2} h^{-1})
Pd/TiO_2-{101}	49	262	5.3
Pd/TiO_2-{100}	94	70	0.74
Pd/TiO_2-{001}	65	24	0.37

Supplementary References

1. Yu, J. et al. Enhanced photocatalytic CO_2 reduction activity of anatase TiO_2 by coexposed {001} and {101} facets. *J. Am. Chem. Soc.* **136**, 8839-8842 (2014).
2. Li, L. et al. Efficient sunlight-driven dehydrogenative coupling of methane to ethane over a Zn^{+}-modified zeolite. *Angew. Chem. Int. Ed.* **50**, 8299-8303 (2011).
3. Li, L. et al. Synergistic effect on the photoactivation of the methane C−H bond over Ga^{3+}-modified ETS-10. *Angew. Chem. Int. Ed.* **51**, 4702-4706 (2012).
4. Meng, L. et al. Gold plasmon-induced photocatalytic dehydrogenative coupling of methane to ethane on polar oxide surfaces. *Energy Environ. Sci.* **11**, 294-298 (2018).
5. Yu, L., Shao, Y. & Li, D. Direct combination of hydrogen evolution from water and methane conversion in a photocatalytic system over Pt/TiO_2. *Appl. Catal. B Environ.* **204**, 216-223 (2017).
6. Wu, S. et al. Ga-doped and Pt-loaded porous TiO_2−SiO_2 for photocatalytic nonoxidative coupling of methane. *J. Am. Chem. Soc.* **141**, 6592-6600 (2019).
7. Yu, X. et al. Stoichiometric methane conversion to ethane using photochemical looping at ambient temperature. *Nat. Energy* **5**, 511-519 (2020).
8. Jiang, W. et al. Pd-modified ZnO-Au enabling alkoxy intermediates formation and dehydrogenation for photocatalytic conversion of methane to ethylene. *J. Am. Chem. Soc.* **143**, 269-278 (2021).
9. Chen, Z. et al. N-type doping induced electron localization for non-oxidative coupling of methane. *Angew. Chem. Int. Ed.* **60**, 11901-11909 (2021).
10. Zhang, L. et al. Visible-light-driven non-oxidative dehydrogenation of alkanes at ambient conditions. *Nat. Energy* **7**, 1042-1051 (2022).
11. Zhang, W. et al. High-performance photocatalytic nonoxidative conversion of methane to ethane and hydrogen by heteroatoms-engineered TiO_2. *Nat. Commun.* **13**, 2806 (2022).
