# Supplementary material for: Unusual facet and co-catalyst effects in TiO2-based photocatalytic coupling of methane
Source: Nat Commun. 2024 May 24;15:4453. doi: 10.1038/s41467-024-48866-1 (PMC11126583; doi:10.1038/s41467-024-48866-1)
Supplement: Supplementary file 3 — Reporting Summary [file 41467_2024_48866_MOESM3_ESM.pdf]

nature portfolio

Corresponding author(s): Ye Wang

Last updated by author(s): Apr 26, 2024

Reporting Summary

Nature Portfolio wishes to improve the reproducibility of the work that we publish. This form provides structure for consistency and transparency in reporting. For further information on Nature Portfolio policies, see our Editorial Policies and the Editorial Policy Checklist.

Statistics

For all statistical analyses, confirm that the following items are present in the figure legend, table legend, main text, or Methods section.

n/a	Confirmed	
☒	☐	The exact sample size (*n*) for each experimental group/condition, given as a discrete number and unit of measurement
☐	☒	A statement on whether measurements were taken from distinct samples or whether the same sample was measured repeatedly
☒	☐	The statistical test(s) used AND whether they are one- or two-sided<br>*Only common tests should be described solely by name; describe more complex techniques in the Methods section.*
☒	☐	A description of all covariates tested
☒	☐	A description of any assumptions or corrections, such as tests of normality and adjustment for multiple comparisons
☒	☐	A full description of the statistical parameters including central tendency (e.g. means) or other basic estimates (e.g. regression coefficient) AND variation (e.g. standard deviation) or associated estimates of uncertainty (e.g. confidence intervals)
☒	☐	For null hypothesis testing, the test statistic (e.g. *F*, *t*, *r*) with confidence intervals, effect sizes, degrees of freedom and *P* value noted<br>*Give P values as exact values whenever suitable.*
☒	☐	For Bayesian analysis, information on the choice of priors and Markov chain Monte Carlo settings
☒	☐	For hierarchical and complex designs, identification of the appropriate level for tests and full reporting of outcomes
☒	☐	Estimates of effect sizes (e.g. Cohen's *d*, Pearson's *r*), indicating how they were calculated

Our web collection on statistics for biologists contains articles on many of the points above.

Software and code

Policy information about availability of computer code

Data collection	The theoretical calculations are performed with VASP 6.3 software and Gaussian 16 software.
Data analysis	The data are plotted into Figures with Origin Pro 8.5 edition.

For manuscripts utilizing custom algorithms or software that are central to the research but not yet described in published literature, software must be made available to editors and reviewers. We strongly encourage code deposition in a community repository (e.g. GitHub). See the Nature Portfolio guidelines for submitting code & software for further information.

Data

Policy information about availability of data

All manuscripts must include a data availability statement. This statement should provide the following information, where applicable:

- Accession codes, unique identifiers, or web links for publicly available datasets
- A description of any restrictions on data availability
- For clinical datasets or third party data, please ensure that the statement adheres to our policy

All data that support the findings in the current study are available within the Article and its Supplementary Information. Source data are available from the corresponding authors upon reasonable request.

Research involving human participants, their data, or biological material

Policy information about studies with human participants or human data. See also policy information about sex, gender (identity/presentation), and sexual orientation and race, ethnicity and racism.

Reporting on sex and gender	Human research participants and Sex and Gender in research were not included in this study.
Reporting on race, ethnicity, or other socially relevant groupings	Race, ethnicity, or other socially relevant groupings in research were not included in this study.
Population characteristics	See above
Recruitment	See above
Ethics oversight	See above

Note that full information on the approval of the study protocol must also be provided in the manuscript.

Field-specific reporting

Please select the one below that is the best fit for your research. If you are not sure, read the appropriate sections before making your selection.

☐ Life sciences ☐ Behavioural & social sciences ☒ Ecological, evolutionary & environmental sciences

For a reference copy of the document with all sections, see nature.com/documents/nr-reporting-summary-flat.pdf

Ecological, evolutionary & environmental sciences study design

All studies must disclose on these points even when the disclosure is negative.

Study description	Pd modified TiO2 catalysts were fabricated by an adsorption-reduction method for catalyzing methane coupling in the presence of water under irradiation.
Research sample	The research samples are mainly focused on Pd modified TiO2 nanocrystals.
Sampling strategy	TEM and HRTEM were conducted to measure the of the size and morphologies of the resultant materials.
Data collection	H.Z. performed most of the experiments and data analysis. P.S. conducted theoretical calculations and analysed the data. X.F., Z.H., W.Z., Q.G., and Y.Z. performed characterizations of photocatalysts and photocatalytic processes and analysed the data.
Timing and spatial scale	The start and stop dates of data collection were Dec. 6, 2020 and Feb. 20, 2024, respectively. From product preparation to characterization of physical properties and the subsequent application testing, the overall frequency and periodicity of sampling were about once a month.
Data exclusions	No data were excluded from the analyses.
Reproducibility	Exact numbers for each individual type of experiment are provided in Figure legends and in the Source Data file. All attempts to repeat the experiment were successful.
Randomization	Solutions containing samples were randomly added dropwise onto the Cu grids. The corresponding particles were randomly selected in TEM and HRTEM grids.
Blinding	Investigators were not blinded to group allocation during data collection and analysis due to limited operators and funds budget.

Did the study involve field work? ☐ Yes ☒ No

Reporting for specific materials, systems and methods

We require information from authors about some types of materials, experimental systems and methods used in many studies. Here, indicate whether each material, system or method listed is relevant to your study. If you are not sure if a list item applies to your research, read the appropriate section before selecting a response.

Materials & experimental systems

n/a	Involved in the study
☒	☐ Antibodies
☒	☐ Eukaryotic cell lines
☒	☐ Palaeontology and archaeology
☒	☐ Animals and other organisms
☒	☐ Clinical data
☒	☐ Dual use research of concern
☒	☐ Plants

Methods

n/a	Involved in the study
☒	☐ ChIP-seq
☒	☐ Flow cytometry
☒	☐ MRI-based neuroimaging

Plants

Seed stocks	Plants in research was not included in this study.
Novel plant genotypes	Plants in research was not included in this study.
Authentication	Plants in research was not included in this study.
